# How does damage control strategy influence organ’s suitability for donation after major trauma? A multi-institutional study

**DOI:** 10.1007/s00068-024-02488-w

**Published:** 2024-04-09

**Authors:** Michele Altomare, Shir Sara Bekhor, Marco Sacchi, Federico Ambrogi, Gabriele Infante, Arturo Chieregato, Federico Pozzi, Tullia Maria De Feo, Lorenza Nava, Elisabetta Masturzo, Luca Del Prete, Carolina Perali, Elena Manzo, Paolo Bertoli, Francesco Virdis, Andrea Spota, Stefano Piero Bernardo Cioffi, Laura Benuzzi, Giuliano Santolamazza, Mauro Podda, Andrea Mingoli, Osvaldo Chiara, Stefania Cimbanassi

**Affiliations:** 1 Department of Surgical Sciences, Sapienza University of Rome, Piazzale Aldo Moro 5, 00185 Rome, Italy; 2 Department of Acute Care Surgery and Trauma Team, ASST GOM Niguarda, Piazza Ospedale Maggiore 3, Milano, 20162 Milan, Italy; 3 Department EMERGENZA, URGENZA-E.A.S. SOREU Metropolitana, Via Campanini 6, 20124 Milan, Italy; 4 Department of Clinical Science and Community Health, University of Milan, Festa del Perdono 7, 20122 Milan, Italy; 5 Department of Economics, Management and Quantitative Methods (DEMM), University of Milan, Via Festa del Perdono 7, Milan, Italy; 6 SSD Clinical Epidemiology and Trial Organization, Department of Epidemiology and Data Science, Fondazione IRCCS Istituto Nazionale dei Tumori, via Venezian 1, Milan, Italy; 7 Neuro-intensive Care Unit, ASST Niguarda Hospital, Piazza Ospedale Maggiore 3, 20162 Milan, Italy; 8 UOC Trapianti Lombardia - NITp Fondazione IRCCS Ca’, Granda Ospedale Maggiore Policlinico Milano, Milan, Italy; 9 SS Coordinamento locale del prelievo di Organi e Tessuti, ASST Niguarda, Piazza Ospedale Maggiore 3, 20162 Milan, Italy; 10 General and Liver Transplantation Surgery Unit, Fondazione IRCCS Ca’ Granda, Ospedale Maggiore Policlinico, Via Francesco Sforza 28, 20122 Milan, Italy; 11 ASST Fatebenefratelli Sacco, Piazza Principessa Clotilde 3, 20157 Milan, Italy; 12 ASST Papa Giovanni XXIII, Piazza OMS – Organizzazione Mondiale della Sanità 1, 20147 Bergamo, Italy; 13 General Surgery Residency Program, University of Milan, Festa del Perdono 7, 20122 Milan, Italy; 14 Department of Surgical Science, Emergency Surgery Unit, University of Cagliari, Cagliari, Italy; 15 Department of Medical-Surgical Physiopathology and Transplantation, University of Milan, Festa del Perdono 7, 20122 Milan, Italy

**Keywords:** Trauma Donors, Organ donation, Major trauma, Damage control surgery, Damage control strategy, Damage control

## Background

Advancements in immunology, surgical techniques, and technology have made solid organ transplantation a routine life-saving treatment for organ failure since its introduction in the 1950s [1]. Organ availability has always been a significant setback, and the gap between organ supply and demand continues to increase as the number of patients added to waiting lists rises. In 2021, over 100,000 patients were on waiting lists for organ transplantation in the USA [2] and about 48,000 in Europe [3]. The lack of suitable organs leads to longer waiting times and increased morbidity and mortality among these patients. To overcome this shortage, efforts towards possible means to increase the available donor pool are being made. While ongoing research attempts to identify other organ sources for transplantation, from xenografts [4] to lab-grown organs [5], the vast majority (about 70%) of donated organs come from deceased donors, of which about 30% are trauma patients [6]. Previous studies identified that trauma donors (TD) are younger, have fewer comorbidities, yield more organs per donor, and are more likely to produce extra-renal organs [7]. Current practices that have somewhat contributed to the increase in organ procurement from TD include the expanded criteria donors [8] and donation after circulatory death (DCD) [9]. In front of a severely injured patient, the physician’s primary focus must be the patient’s well-being. However, when the patient presents with nonsurvivable injuries, resuscitation for future organ donation should be kept in mind. Currently, there are no established guidelines to follow in this field, and trauma surgeons face an ethical dilemma of deciding whether, when, and how to resuscitate a patient who may not directly benefit from it [10]. Considering that practices of trauma management may vary regionally, it may be helpful to understand whether and how these practices affect organ donation. In the management of severe trauma patients, according to the mechanism of injury and degree of physiologic derangement, damage control strategies (DCS) may be applied. DCS is a compilation of surgical and medical techniques that focus on the rapid correction of altered and deteriorating physiology, control of hemorrhage and contamination, and resuscitation of critical patients. These strategies have the potential to decrease mortality, improve organ perfusion, and increase organ survival after brain death declaration. Understanding the relationship between DCS and outcomes of organ donation (in terms of both quantity and quality) can potentially contribute to increasing the available organ pool without compromising resuscitation attempts of severe trauma patients. So far, few case reports have been published regarding organ donation after damage control laparotomy or abdominal decompression, suggesting that aggressive resuscitation of severe trauma patients has increased the number of organs available for transplantation [11–13]. A series published by our team provided initial insight into the characteristics of organ donation after DCS [14]. However, to our knowledge, no statistical models nor comparative studies have been published on the subject to date. Keeping in mind the importance of thoroughly exploring such a nuanced topic as the potential futility of treatment in polytrauma patients, the hypothesis underlying this study is to try to understand whether the failure of the damage control strategy, when failing in its primary goal of sustaining the life of the polytrauma patient, can at least assist the trauma surgeon in saving potentially donatable organs.

Therefore, the primary aim of this study is to characterize subtypes of organ donors after major trauma and examine a possible relationship between the application of DCS and organ donation outcomes within an Italian population.

## Methods

An observational cohort study on trauma donors was conducted through the revision of data from three major hospitals in Northern Italy. The STROBE statement checklist for the cohort study was used to report the data. Detailed characteristics of the hospitals are reported in the supplementary materials 1. All three hospitals hold both a Level I/II trauma center and a transplant center, part of the national network formed by the National Transplant Center (*Centro Nazionale Trapianti*, CNT). Data search and collection from electronic patient records identified death brain donors (DBD) whose cause of death was major trauma from January 2012 to September 2021. From September 2021 to March 2022, data collection continued prospectively.

Inclusion criteria were as follows: patient admitted to the hospital by emergency services following major trauma (red code); at least one solid organ procured and transplanted (considering heart, lungs, liver, kidneys, and pancreas); availability of records from pre-hospital care, ED admission and patient diary from the ICU; attainability of data regarding the donated organs’ short-term functional outcome. Information regarding the outcomes of transplanted organs was extracted from the Northern Italy Transplant System, NIT.

### Data management and machine learning cluster analysis

Due to the natural variability of subjects and to better characterize trauma donors, to reduce the variability between patients, and to better categorize the patient’s characteristics, the grouping was performed by a machine learning cluster analysis according to trauma-related clinical and laboratory variables. A hierarchical algorithm was used for clustering the patients and selected variables producing a graph called a heatmap. The heatmap (Fig. 2) is a visual representation of how the different clusters of patients differ according to the variables used for the clustering itself. The algorithm groups objects based on their similarity, calculated using Euclidean distance. The algorithm starts by treating each object as a single-element cluster, and pairs of elements are successively merged until all clusters have been grouped into a single large one containing all objects. Therefore, this method is defined as bottom-up clustering. Finally, objects and/or clusters in close proximity are linked via the linkage function, which takes the distance information and groups pairs of objects into clusters to create bigger ones. When using hierarchical methods, possible linkage functions are average, single, complete, and ward.

To determine the optimal number of clusters, the elbow, silhouette, and gap statistics methods were taken into consideration [15]. The Elbow method relies on the inspection of the sum of squared distances between each object and the centroid of a cluster (within-cluster sum of squares, WCSS). When the number of clusters increases, the WCSS decreases. Usually, there is a fast decrease at the beginning, and then, starting from a certain number of clusters, a linear decrease is observed. The suggested number of clusters corresponds to the end of the fast decrease (the “elbow”). The silhouette index measures how well an observation is clustered and estimates the average distance between clusters. The overall average silhouette width, which is the average of the silhouettes for all patients, is used to compare clustering solutions. Larger values suggest a stronger clustering structure. Lastly, the gap statistics method measures how much the clustering solution is different from the uniform distribution of the values where no clusters are present (null hypothesis). The number of clusters that maximizes the gap statistics is the suggested number of groups.

The variables included in the cluster formation were patient data and trauma-related variables from pre-hospital and emergency department (ED) settings. In particular, patient data included: age, gender, and BMI. Pre-hospital variables included: systolic blood pressure (Pre_SBP), heart rate (Pre_HR), shock index (Pre_SI), Glasgow Coma Scale (Pre_GCS), and cardiac arrest (Pre_CA). Emergency department variables included the following: systolic blood pressure (ED_SBP), heart rate (ED_HR), Glasgow Coma Scale (ED_GCS), cardiac arrest at admission (ED_CA), shock index (ED_SI), the difference between Pre_SI and ED_SI (delta_SI), and arterial blood gas analysis findings (lactate, base excess, pH). In addition, organ injuries were classified using the Abbreviated Injury Scale (AIS) 2015 revision; injury severity score (ISS) was calculated accordingly and included in the analysis.

DCS procedures registered in the study were all the procedures performed on patients to temporarily or permanently stabilize the hemodynamic: monolateral/bilateral decompressive thoracostomies, ED thoracotomy, extraperitoneal pelvic packing (EPP), REBOA positioning, exploratory laparotomy, exploratory thoracotomy, decompressive craniotomy, external bone fixation, therapeutic angiography with embolization. Damage control resuscitation registered in the study were as follows: tranexamic acid (TXA) administration, vasoactive drugs use, massive transfusion protocol (MTP) activation, and ED crystalloid administration (pre-hospital crystalloids were not considered since it is administered almost by default in the injury scene). ICU management of trauma donors before and after brain death declaration was not included in the study since it is generally standardized.

Next, donation and transplantation of the heart, lungs, liver, and kidneys, were registered for each subject. The short-term functional outcome of the transplanted organs was evaluated in terms of 30-day graft dysfunction requiring explantation. The functional response rate was defined as the proportion of organs with a positive functional outcome from total transplanted organs.

### Statistical methods

Normality and heteroskedasticity of continuous data were assessed with Shapiro–Wilk and Levene’s tests, respectively. Continuous variables were compared with ANOVA or Kruskal Wallis tests according to data distribution. Categorical variables were compared with chi-squared or Fisher’s exact test accordingly. Variables were expressed as mean ± SD or relative frequencies (%). The type I error was set to 5%, and two-tailed tests were used. A generalized linear model was used to evaluate the association of clusters with the probability of organ donation to consider possible “confounding” factors such as age. Statistical analysis was performed with EasyMedStat (version 3.18; www.easymedstat.com) and R free statistical software (www.r-project.org/).

## Results

**Fig. 1:**
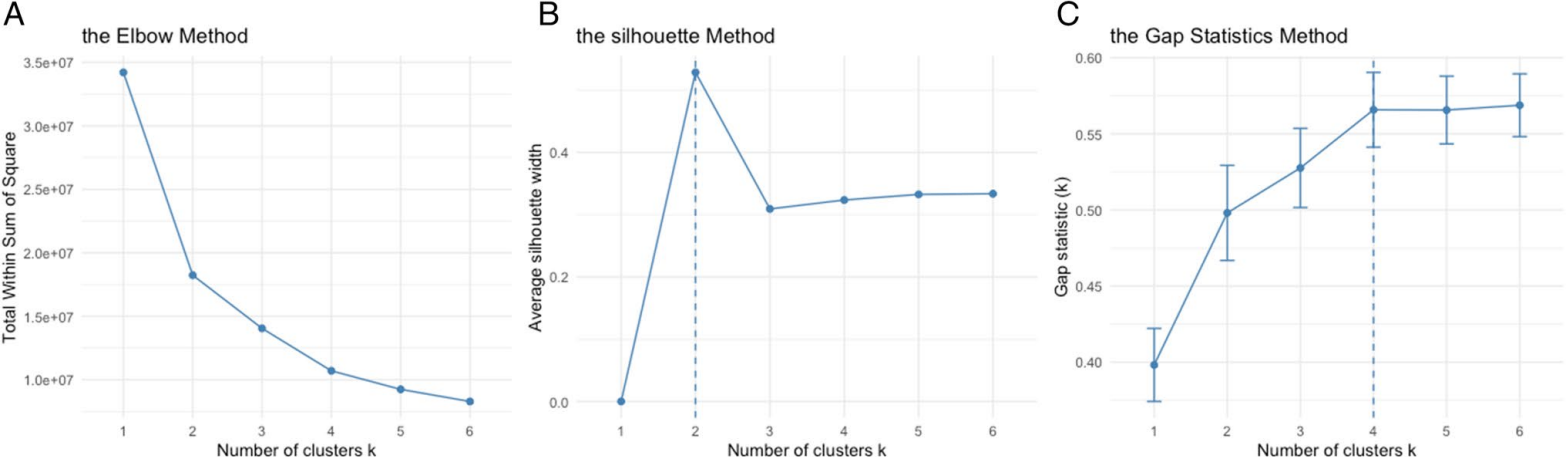
**A** According to the elbow method, the optimal number of clusters is two. **B** According to the silhouette method, the optimal number of clusters is two. **C** According to the gap statistics method, the optimal number of clusters is four

Data search from the abovementioned hospitals identified 124 organ donors whose cause of death was major trauma. Of these, seven (5%) were excluded for incomplete data. Following data collection and cleaning, multivariate analysis by clustering heatmap was performed using the selected variables. To determine the optimal number of clusters, three methods were compared, as demonstrated in Fig. 1.

**Fig. 2:**
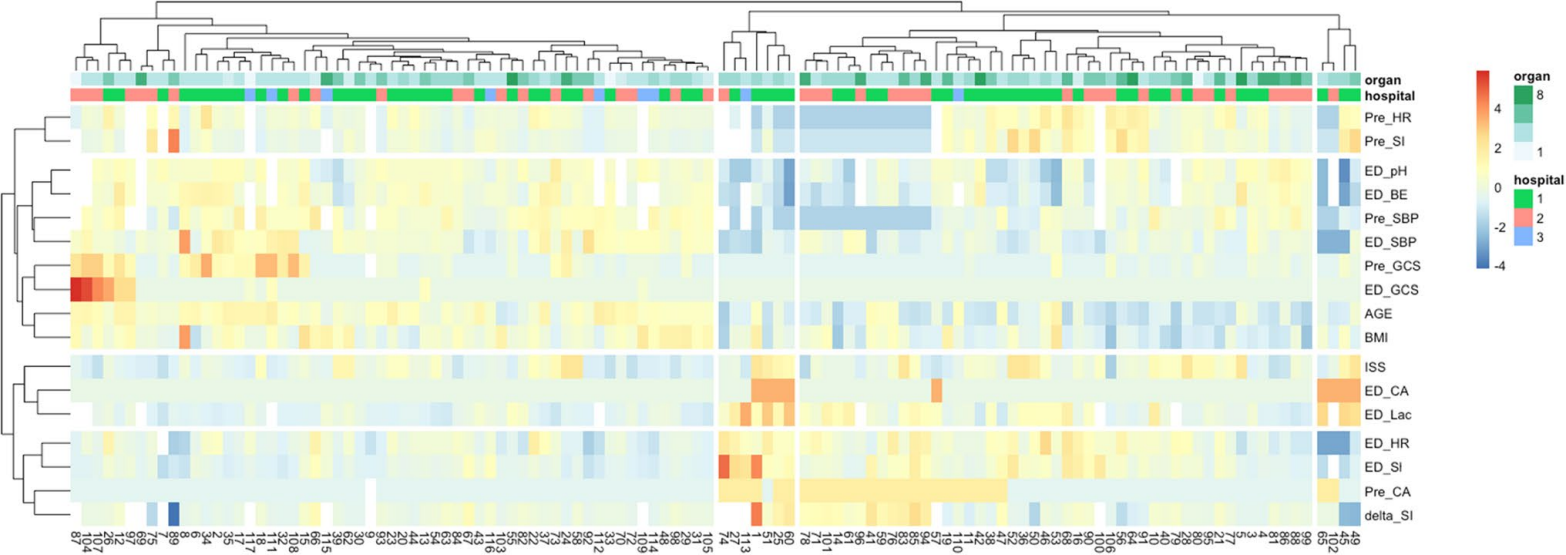
Clustering heatmap of 117 trauma donors has identified two main clusters. Pre_HR, pre-hospital heart rate; Pre_SI, pre-hospital shock index; ED_pH, pH level measured in the emergency department; ED_BE, base excess levels measured in the emergency department; Pre_SBP, pre-hospital systolic blood pressure; ED_SBP, emergency department systolic blood pressure; Pre_GCS, pre-hospital Glasgow Coma Scale; ED_GCS, emergency department Glasgow Coma Scale; BMI, body mass index; ISS, injury severity score; ED_CA, cardiac arrest at admission to emergency department; ED_Lac, lactate levels measured in emergency department; ED_HR, emergency department heart rate; ED_SI, emergency department shock index; Pre_CA, pre-hospital cardiac arrest; delta_SI, difference between pre-hospital and emergency department shock index. Organ: total number of donated organs, from 1 to 8 (including heart, lungs, liver, kidneys, and pancreas). Hospital: 1, Niguarda; 2, Papa Giovanni; 3, Policlinico

According to the elbow method and the silhouette index, two clusters could be considered. According to the gap statistics, four clusters can be identified. By observation of the heatmap, it is possible to note two main clusters, one of which can be further divided into three subgroups [15] (Fig. 2).

The two main clusters are composed of almost an equal number of patients (Cluster 1 is made of 58 subjects vs. 59 subjects in Cluster 2). They do not differ in patient distribution by hospitals (33 vs. 32 from Niguarda, 23 vs. 20 from Papa Giovanni, and 2 vs. 7 from Policlinico, *p* = 0.224). Regarding patient-related variables included in the analysis, the clusters do not differ in gender distribution; there are 41 males in Cluster 1 and 44 males in Cluster 2 (70% vs. 74%, *p* = 0.792). However, the clusters significantly differ in patient age (29.39 ± 18.87 vs. 61.26 ± 14.36, *p* < 0.001) and BMI (22.8 ± 3.66 vs. 26.43 ± 3.62, *p* < 0.001). As for pre-hospital and emergency department trauma-related variables, the clusters significantly differ in Pre_SBP (70.04 ± 58.06 vs. 138.9 ± 35.78, *p* < 0.001), Pre_GCS (3.41 ± 0.879 vs. 5.88 ± 3.62, *p* < 0.001), Pre_CA (46.55% vs. 0%, *p* < 0.001), ED_SBP (91.76 ± 35.72 vs. 136.52 ± 37.59, *p* < 0.001), ED_HR (104.47 ± 36.81 vs. 87.09 ± 23.87, *P* < 0.001), ED_GCS (3.0 ± 0 vs. 3.88 ± 2.45, *p* = 0.001), ED_CA (15% vs. 0%, *p* = 0.001), and ED_SI (1.27 ± 0.746 vs. 0.683 ± 0.303, *p* < 0.001). However, there are no significant differences in Pre_HR (73.06 ± 57.55 vs. 86.94 ± 26.9, *p* = 0.527) and Pre_SI (0.784 ± 0.733 vs. 0.716 ± 0.479, *p* = 0.678). The clusters significantly differ in the laboratory arterial-blood gas analysis variables that were examined: pH (7.12 ± 0.232 vs. 7.28 ± 0.11, *p* < 0.001), base excess (− 11.6 ± 7.36 vs. − 4.86 ± 4.33, *p* < 0.001), and lactate (7.09 ± 4.49 vs. 3.18 ± 1.6, *p* < 0.001).

The last variable used to characterize the clusters was ISS, which significantly differs between the clusters (42.93 ± 17.38 vs. 34.19 ± 13.27, *p* = 0.002). We can identify the source of this difference by looking into the abbreviated injury score (AIS) of each solid organ of interest. In Cluster 1, seven (12%) livers were classified as AIS ≥ 2, while all Cluster 2 livers were uninjured, meaning AIS of zero (*p* = 0.006). The same is true for pancreas injury; in Cluster 1, five (8%) were injured with AIS ≥ 1, while in Cluster 2, none were injured (*p* = 0.027). Lung, traumatic injuries of AIS ≥ 2 were quite common in both groups yet more prevalent in Cluster 1 (72% vs. 45%, *p* = 0.004). No significant difference regarding heart injuries (0% vs. 1.69%, *p* > 0.999) nor kidney injuries (8% vs. 5%, *p* = 0.458) were found. The brain injury severity was similar between the clusters (4.54 ± 1.16 vs. 4.81 ± 0.473, *p* = 0.293).

A significant difference between the clusters was found with regard to the total number of DCS procedures applied (4.31 ± 2.54 vs. 1.98 ± 1.54, *p* < 0.001). In particular, more subjects in Cluster 1 had DCS applied already in the injury scene (62% vs. 11%, *p* < 0.001). Looking into the damage control resuscitation maneuvers considered in this study, more Cluster 1 patients received vasoactive agents both in the pre-hospital settings (40% vs. 5%, *p* < 0.001) and in the ED (58% vs. 23%, *p* < 0.001). The same is true for tranexamic acid in the pre-hospital settings (38% vs. 8%, *p* < 0.001) and in the ED (43% vs. 18%, *p* = 0.008). More Cluster 1 patients were administered with crystalloids in the ED (76% vs. 59%, *p* = 0.024) and required activation of massive transfusion protocol (37% vs. 16%, *p* = 0.019). Regarding damage control surgeries (EPP, thoracostomy, laparotomy, thoracotomy, craniotomy, ODC, and angiography), the average number of applied procedures was significantly higher in Cluster 1 (1.67 ± 1.8 vs. 0.83 ± 0.91, *p* = 0.004).

With regard to the donation of solid organs, Cluster 1 has produced significantly more hearts (65% vs. 34%, *p* = 0.001). Almost all subjects were liver donors with no significant difference between clusters (96% vs. 94%, *p* > 0.999). However, Cluster 1 produced more split livers (22% vs. 3%, *p* = 0.002). No significant difference regarding lung donation (29% vs. 15%, *p* = 0.108), kidney donation (96% vs. 90%, *p* = 0.272), or pancreas donation (29% vs. 27%, *p* = 0.954) was found. On average, Cluster 1 donated more organs per donor than Cluster 2 (4.5 ± 1.62 vs. 3.59 ± 1.43, *p* = 0.001). Interestingly, the functional response rate, defined as the proportion of organs that did not have primary dysfunction in the first 30 days from all transplanted organs, was equal (93% vs. 93%, *p* = 0.929).

**Fig. 3:**
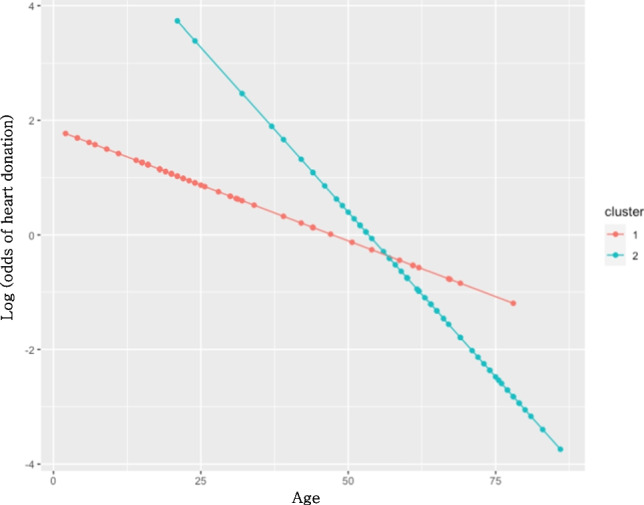
Logistic regression of heart donation (dependent variable) as a function of age and cluster (independent variables) demonstrating that the effect of age is different in each cluster

A logistic regression model of heart donation as a function of cluster and age was performed further to investigate the effect of age on organ donation. The estimation results showed a significant interaction between age and cluster (*p* = 0.04). Figure 3 shows the probability of heart donation as a function of age for the two clusters. It is noticeable that this probability decreases more rapidly in Cluster 2 (characterized by a higher mean age) than in Cluster 1 (characterized by a lower mean age). For example, comparing a 40-year-old patient to a 50-year-old patient, the odds ratio of heart donation is 3.16 (95% CI 1.65–6.07) in Cluster 2 and 1.48 (95% CI 1.08–2.01) in Cluster 1.

## Discussion

Worldwide, the main source of organs for transplantation is deceased donors, especially following brain death. As already established in the literature, among deceased donors, those who pass following major trauma are younger and have fewer comorbidities [7], as intuitively associated with the epidemiology of trauma. More importantly, it has been demonstrated that trauma donors produce more solid organs other than kidneys and exhibit lower (better) Kidney Donor Risk Index scores, meaning that trauma donors make an important contribution to the donor pool. From our experience, trauma patients are highly heterogeneous, some of which call for the investment of important resources in an aggressive attempt at stabilization and resuscitation. Since there are no strict guidelines to follow, oftentimes, it is the trauma team leader’s responsibility to decide whether and how much of these resources to use, considering all possible outcomes. For this reason, we have decided to study trauma donors and look for possible relationships between their initial clinical state and management to their outcomes in terms of the quantity and quality of donated organs. With the use of machine learning heatmap clustering, we were thus able to identify two main sub-groups of trauma donors; the first (Cluster 1) includes younger patients who had severe, multidistrict traumatic injuries associated with hemodynamic instability and physiologic alteration requiring aggressive resuscitation attempts with surgical and medical means. The second (Cluster 2) includes mainly older patients who mostly suffered an isolated traumatic brain injury, with no major alterations in hemodynamic parameters calling for DCS (but the occasional decompressive craniotomy as an attempt to improve neurological status). Interestingly, although the first cluster is composed of patients who suffered greater multi-system injuries, cardiac arrests, and hypotension, they have donated more solid organs with respect to the second. This may be related to the fact that donors in this cluster are younger, yet the circumstances preceding the declaration of death should not be taken for granted.

Regional transplant centers have varying absolute and relative exclusion criteria for potential organ donors [16], in some of which prolonged hypotension, hypothermia, and coagulopathy may be contraindications to organ donation since these conditions may increase the risk of primary non-function [17]. This study’s findings suggest that with the application of DCS, which aims for the correction of these alterations, adequate organ perfusion may be restored, allowing for organ preservation. In our experience, it appears that DCS contributes to the salvage of injured organs that would have otherwise been discarded. For example, six injured livers, all AIS 2 and above, were successfully transplanted with good short-term functional outcomes [18], all following management with an aggressive DCS approach. In addition, eight pairs of lungs with AIS of 2 or 3 were donated and transplanted. One lung was donated following an injury of AIS 4.

Our findings support Elmer et al. (2019), who claimed that patients resuscitated from cardiac arrest with irrecoverable brain injury have excellent potential to become organ donors [19]. In fact, a total of 15 hearts were successfully transplanted after being resuscitated from cardiac arrest in the injury scene and/or shock room, all from Cluster 1 (and, interestingly, all from Level I trauma centers Niguarda and Papa Giovanni). The most outstanding outcome is that 65% of Cluster 1 subjects were heart donors, especially considering the scarcity of this resource. This outcome could not be solely explained by the difference in age and might be the topic of future investigations.

The main benefit of this paper is an illustration of the fact that aggressive resuscitation and invasive procedures do not preclude successful organ donation for those patients and families who choose to donate. This information could minimize hesitance to perform DCS interventions in patients with non-survivable brain injuries, which is important not only to preserve the option of organ donation for those patients and families who choose it but also to maximize the chances of survival for those patients with severe injuries who do have a chance to recover.

The novelty of our study, other than the use of machine learning and clustering, is taking a step back to examine these donors at a time when the primary aim is their stabilization and resuscitation as traumatic patients.

On the other hand, there are several limitations to this study which mainly derive from its retrospective nature and the use of electronic medical records. Trauma management is highly dynamic, and the interpretation of events depends on the accuracy of their description and the level of detail from both pre-hospital and in-hospital patient records. The relative scarcity of each particular DCS intervention considered in the study does not permit discussing their relation to organ donation. Thus, they were discussed as a group. Moreover, we acknowledge that our definition of functional response considering 30 days post-transplant is relatively short-term and was chosen due to the attainability of data.

Moreover, the pathway involving consent, procurement, and the outcome leading to successful organ donation is intricate and impacted by numerous non-clinical factors. These encompass advanced directives, the preferences of the family, religious beliefs, racial aspects, and biases held by both trauma and transplant surgeons regarding the suitability of specific organs for donation. This study exclusively focuses on clinical factors, although numerous; therefore, it might not comprehensively capture the entirety of successful organ donation dynamics.

In conclusion, we showed that an aggressive DCS to save trauma patients’ lives does not negatively impact the chances of organ donation in suitable donors, producing a significant number of organs for transplantation with good functional outcomes. Our data may contribute to highlighting how organ donation can be considered a relevant outcome in severely injured trauma patients in which survival cannot be guaranteed after maximal resuscitation efforts.

Supplementary information.

## Supplementary Information

Below is the link to the electronic supplementary material.

Supplementary file1 (DOCX 13 KB)

## Data Availability

No datasets were generated or analyzed during the current study.
