# Publisher Correction: How does damage control strategy influence organ's suitability for donation after major trauma? A multi-institutional study

**DOI:** 10.1007/s00068-024-02593-w

**Published:** 2024-09-17

**Authors:** Michele Altomare, Shir Sara Bekhor, Marco Sacchi, Federico Ambrogi, Gabriele Infante, Arturo Chieregato, Federico Pozzi, Tullia Maria De Feo, Lorenza Nava, Elisabetta Masturzo, Luca Del Prete, Carolina Perali, Elena Manzo, Paolo Bertoli, Francesco Virdis, Andrea Spota, Stefano Piero Bernardo Cioffi, Laura Benuzzi, Giuliano Santolamazza, Mauro Podda, Andrea Mingoli, Osvaldo Chiara, Stefania Cimbanassi

**Affiliations:** 1 Department of Surgical Sciences, Sapienza University of Rome, Piazzale Aldo Moro 5, 00185 Rome, Italy; 2 Department of Acute Care Surgery and Trauma Team, ASST GOM Niguarda, Piazza Ospedale Maggiore 3, Milano, 20162 Milan, Italy; 3 Department EMERGENZA, URGENZA-E.A.S. SOREU Metropolitana, Via Campanini 6, 20124 Milan, Italy; 4 Department of Clinical Science and Community Health, University of Milan, Festa del Perdono 7, 20122 Milan, Italy; 5 Department of Economics, Management and Quantitative Methods (DEMM), University of Milan, Via Festa del Perdono 7, Milan, Italy; 6 SSD Clinical Epidemiology and Trial Organization, Department of Epidemiology and Data Science, Fondazione IRCCS Istituto Nazionale dei Tumori, via Venezian 1, Milan, Italy; 7 Neuro-intensive Care Unit, ASST Niguarda Hospital, Piazza Ospedale Maggiore 3, 20162 Milan, Italy; 8 UOC Trapianti Lombardia - NITp Fondazione IRCCS Ca’, Granda Ospedale Maggiore Policlinico Milano, Milan, Italy; 9 SS Coordinamento locale del prelievo di Organi e Tessuti, ASST Niguarda, Piazza Ospedale Maggiore 3, 20162 Milan, Italy; 10 General and Liver Transplantation Surgery Unit, Fondazione IRCCS Ca’ Granda, Ospedale Maggiore Policlinico, Via Francesco Sforza 28, 20122 Milan, Italy; 11 ASST Fatebenefratelli Sacco, Piazza Principessa Clotilde 3, 20157 Milan, Italy; 12 ASST Papa Giovanni XXIII, Piazza OMS – Organizzazione Mondiale della Sanità 1, 20147 Bergamo, Italy; 13 General Surgery Residency Program, University of Milan, Festa del Perdono 7, 20122 Milan, Italy; 14 Department of Surgical Science, Emergency Surgery Unit, University of Cagliari, Cagliari, Italy; 15 Department of Medical-Surgical Physiopathology and Transplantation, University of Milan, Festa del Perdono 7, 20122 Milan, Italy

**Correction to: European Journal of Trauma and Emergency Surgery**

10.1007/s00068-024-02488-w

The original version of this article, published on 09 April 2024, unfortunately contained a mistake.

In this article the title was incorrectly given as 'Multi-centric study on organ donation after trauma: a cluster analysis' but should have been 'How does damage control strategy influence organ's suitability for donation after major trauma? A multi-institutional study'

The following Authors and their affiliations were missing from the author list.

Shir Sara Bekhor

Department of Acute Care Surgery and Trauma Team, ASST GOM Niguarda, Milano, Piazza Ospedale Maggiore 3, 20162 Milan

Marco Sacchi

Department EMERGENZA URGENZA-E.A.S. SOREU Metropolitana, Via Campanini 6, 20124 Milan, Italy

Federico Ambrogi

Department of Clinical Science and Community Health, University of Milan, Festa del Perdono 7, 20122 Milan, Italy

Gabriele Infante

Department of Economics, Management and Quantitative Methods (DEMM), University of Milan, Via Festa del Perdono 7, Milan, Italy

SSD Clinical Epidemiology and Trial Organization; Department of Epidemiology and Data Science, Fondazione IRCCS Istituto Nazionale dei Tumori, via Venezian 1, Milan, Italy

Arturo Chieregato

Neuro-intensive Care Unit, ASST Niguarda Hospital, Piazza Ospedale Maggiore 3, 20162, Milan, Italy

Federico Pozzi

Neuro-intensive Care Unit, ASST Niguarda Hospital, Piazza Ospedale Maggiore 3, 20162, Milan, Italy

Tullia Maria De Feo

UOC Trapianti Lombardia – NITp Fondazione IRCCS Ca', Granda Ospedale Maggiore Policlinico Milano, Milan, Italy

Lorenza Nava

SS Coordinamento locale del prelievo di Organi e Tessuti, ASST Niguarda, Piazza Ospedale Maggiore 3, 20162 Milan, Italy

Elisabetta Masturzo

SS Coordinamento locale del prelievo di Organi e Tessuti, ASST Niguarda, Piazza Ospedale Maggiore 3, 20162 Milan, Italy

Luca Del Prete

General and Liver Transplantation Surgery Unit, Fondazione IRCCS Ca’ Granda, Ospedale Maggiore Policlinico, Via Francesco Sforza 28, 20122 Milan, Italy

Carolina Perali

General and Liver Transplantation Surgery Unit, Fondazione IRCCS Ca’ Granda, Ospedale Maggiore Policlinico, Via Francesco Sforza 28, 20122 Milan, Italy

Elena Manzo

ASST Fatebenefratelli Sacco, Piazza Principessa Clotilde 3, 20157 Milan, Italy

Paolo Bertoli

ASST Papa Giovanni XXIII, Piazza OMS – Organizzazione Mondiale della Sanità 1, 20147

Francesco Virdis

Department of Acute Care Surgery and Trauma Team, ASST GOM Niguarda, Milano, Piazza Ospedale Maggiore 3, 20162 Milan

Andrea Spota

Department of Acute Care Surgery and Trauma Team, ASST GOM Niguarda, Milano, Piazza Ospedale Maggiore 3, 20162 Milan

Stefano Piero Bernardo Cioffi

Department of Surgical Sciences, Sapienza University of Rome, Piazzale Aldo Moro 5, 00185 Rome, Italy

Department of Acute Care Surgery and Trauma Team, ASST GOM Niguarda, Milano, Piazza Ospedale Maggiore 3, 20162 Milan

Laura Benuzzi

General Surgery Residency Program, University of Milan, Festa del Perdono 7, 20122 Milan, Italy

Giuliano Santolamazza

General Surgery Residency Program, University of Milan, Festa del Perdono 7, 20122 Milan, Italy

Mauro Podda

Department of Surgical Science, Emergency Surgery Unit, University of Cagliari, Cagliari, Italy

Andrea Mingoli

Department of Surgical Sciences, Sapienza University of Rome, Piazzale Aldo Moro 5, 00185 Rome, Italy

Osvaldo Chiara

Department of Medical-Surgical Physiopathology and Transplantation, University of Milan, Festa del Perdono 7, 20122 Milan, Italy

Department of Acute Care Surgery and Trauma Team, ASST GOM Niguarda, Milano, Piazza Ospedale Maggiore 3, 20162 Milan

Stefania Cimbanassi

Department of Medical-Surgical Physiopathology and Transplantation, University of Milan, Festa del Perdono 7, 20122 Milan, Italy

Department of Acute Care Surgery and Trauma Team, ASST GOM Niguarda, Milano, Piazza Ospedale Maggiore 3, 20162 Milan

The original article has been corrected.

