# Supplementary material for: How does damage control strategy influence organ’s suitability for donation after major trauma? A multi-institutional study
Source: Eur J Trauma Emerg Surg. 2024 Apr 9;50(5):2281–7. doi: 10.1007/s00068-024-02488-w (PMC11599480; doi:10.1007/s00068-024-02488-w)
Supplement: Supplementary file 1 — Supplementary file1 (DOCX 13 KB) [file 68_2024_2488_MOESM1_ESM.docx]

Supplementary materials 1.

ASST Grande Ospedale Metropolitano Niguarda in Milan (Niguarda) was the first hospital in Italy to apply a model of organized trauma care where general surgeons, anesthesiologists, orthopedic surgeons, and neurosurgeons are available 24 hours a day. In this study, Niguarda Hospital is the coordinating center of a collaboration with two other major hospitals in the Lombardy region. Like Niguarda Hospital, ASST Papa Giovanni XXIII Hospital in Bergamo (Papa Giovanni) is a Level I trauma center, which means it can provide total care for every aspect of injury. IRCCS Ca' Granda Ospedale Maggiore Policlinico in Milano (Policlinico) is a Level II trauma center with the resources necessary to definitively treat trauma, except for some of the specialties. All the three hospital hold a transplant center, part of the national network formed by the National Transplant Center (*Centro Nazionale Trapianti*, CNT).
